# Time pressure and reward value’s influence on visual search performance and behavior: an eye-tracking VR study

**DOI:** 10.1186/s41235-026-00731-z

**Published:** 2026-05-22

**Authors:** Mohamad El Iskandarani, Sara Lu Riggs

**Affiliations:** Systems and Information Engineering Department, University of Virginia, Charlottesville, 22904 VA USA

**Keywords:** Visual search, Reward value, Time pressure, Eye tracking, Virtual reality, Performance

## Abstract

Visual search is shaped by both stress-inducing and motivational factors. Time pressure, a common stressor, can impair performance by prompting hurried search strategies, whereas reward value, a motivator, may enhance performance and efficiency. Although their individual effects are well-established, their joint influence on search performance and behavior is not yet clear—specifically, whether reward value can counteract the performance costs incurred by time pressure, enabling fast yet accurate searches. Moreover, little is known on whether time pressure and reward value exert distinct effects on the two processing components of visual search: target acquisition and target verification. The present eye-tracking study examines: (1) how time pressure and reward value influence search performance and behavior, and (2) whether reward value can counteract the adverse effects of time pressure on performance. Forty participants searched for target objects in a virtual living room under various time pressure and reward value conditions. As expected, time pressure reduced accuracy. However, it incurred longer target acquisition but shorter verification times. In contrast, reward value enhanced target acquisition efficiency and accuracy. It particularly improved accuracy and reduced search time under time pressure, suggesting a mitigating role against the performance decrements incurred by time pressure. Overall, the findings show that time pressure and reward value exert distinct effects on target acquisition and verification, yet their interaction influences performance.

## Significance statement

Everyday tasks often require us to find objects quickly and accurately. Whether a driver must spot the correct highway exit, an emergency responder must find the right instrument, or an air traffic controller must identify the correct signal on a display, visual search is a core component of many tasks. Yet, these searches rarely happen in a vacuum. Rather, they are influenced by stressors, such as time pressure, and motivators, such as the value of the outcome. Understanding how time pressure and reward value jointly impact visual search is therefore crucial for understanding how humans search in real-world scenarios. In the present study, we investigated how time pressure and reward value influenced visual search and performance, with emphasis on the two components of visual search: *target acquisition* (how quickly the target can be located) and *verification* (how long it takes to verify the target). Using virtual reality paired with eye-tracking, we were able to capture search behavior in a naturalistic setting. We found that time pressure induced less thorough search strategies that reduced accuracy, while reward value improved efficiency and could counteract some of the negative effects of time pressure on performance. Ultimately, this work contributes to applications ranging from interface design to training protocols in medicine, transportation, aviation, and military–contexts where the balance between search speed and accuracy can have serious ramifications.

## Introduction

### The attentional processes of visual search

Visual attention comprises a set of cognitive processes that modulate signals within the visual system (Evans et al., 2011). Because the visual environment contains far more information than can be processed simultaneously, one of visual attention’s crucial functions is to filter and select a subset of visual stimuli for further processing. For example, once a subset of visual stimuli is selected, attention supports higher-level operations such as object identification and recognition.

Two modes of attentional control guide visual perception: bottom-up, stimulus-driven factors—such as salience, contrast, or motion—and top-down, goal-directed mechanisms that reflect task demands, expectations, and prior knowledge (Egeth & Yantis, 1997). Bottom-up selection is often characterized as parallel, operating simultaneously across the visual field to generate a priority map of physical salience (Itti & Koch, 2001; Koch, 1987). In contrast, top-down control is commonly described as serial, directing an “attentional spotlight” to individual locations or objects (Connor et al., 2004). Serial selection is closely linked to the sequential deployment of eye movements during visual search. Together, these mechanisms determine which visual locations are prioritized at a given moment (Wolfe & Horowitz, 2017).

Importantly, visual attention can be deployed in two complementary forms: overt and covert attention. Overt attention involves directing the eyes toward a stimulus, thereby aligning the locus of attention with the fovea (Findlay & Gilchrist, 2009; Kinchla, 1992). In contrast, covert attention refers to the allocation of processing resources to a location or object without an accompanying eye movement, enhancing perceptual sensitivity in peripheral regions (Buschman & Miller, 2009). Although these two forms of attention are dissociable, they are also closely interlinked: covert attentional shifts often precede and guide subsequent eye movements, supporting efficient exploration of the visual environment (Rai, 2018).

**Fig. 1:**
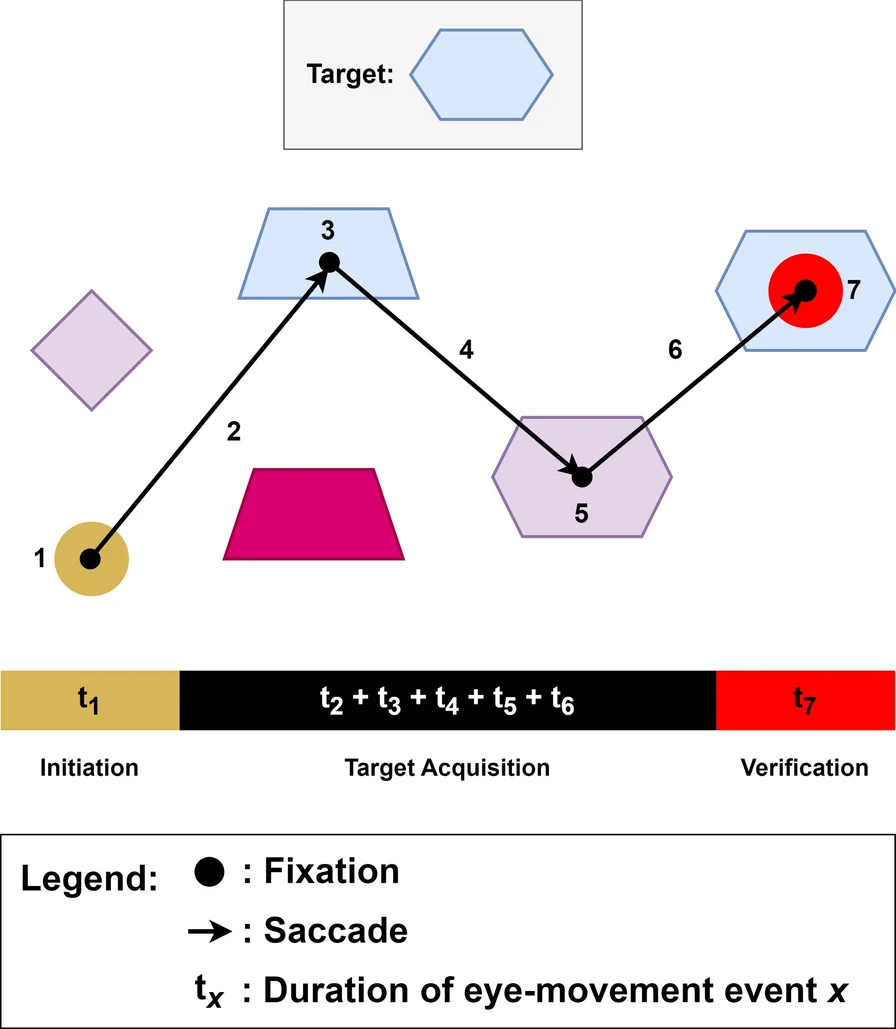
An illustration of the processing components of visual search, inspired by Malcolm and Henderson (2009). The figure depicts initiation and verification as single fixations each for simpler illustration, though each may consist of a series of fixations and saccades

Together, the influence of bottom-up and top-down factors on visual attention, operating through overt or covert shifts, provides the foundation for visual search behavior. Visual search refers to how individuals locate a target among distractors within a scene (Nowakowska et al., 2021; Wolfe, 2020), and it is commonly described as consisting of two processing components illustrated in Fig. 1: target acquisition and verification (Castelhano & Heaven, 2010). Target acquisition spans from the offset of search initiation until the first fixation on the target. It reflects the efficiency of initial guidance and how attention is oriented to relevant stimuli among distractors (Malcolm & Henderson, 2009). Target verification begins once the target is fixated on and covers the subsequent processing needed to determine whether the object matches the intended target. Unlike acquisition, verification engages decision-making and memory processes that finalize the search outcome (Castelhano & Heaven, 2010). Distinguishing between target acquisition and verification allows for a more fine-grained understanding of the attentional processes underlying visual search.

### Visual search and task-related factors

As noted earlier, visual search behavior depends not only on target and scene properties but also on task-related factors that shape search demands and goals (Eckstein, 2011; Hickey & Kaiser, 2015; Wolfe, 2020). Two task-related factors often jointly encountered in real-world search scenarios are time pressure and reward value. Time pressure, defined here as the limited time available to complete a task, often acts as a stressor that can induce faster but potentially less accurate searches (El Iskandarani et al., 2024; El Iskandarani & Riggs, 2023; Rieger & Manzey, 2022; Yu et al., 2015). Reward value, on the other hand, refers to the gain or loss associated with task performance and can guide attention and promote more efficient strategies (Chelazzi et al., 2013; Wolfe, 2020).

The need to investigate time pressure and reward value concurrently is grounded in practical merit, as many everyday searches involve time pressure and reward value simultaneously (Walle & Druey, 2021). For example, emergency responders searching for medical supplies in a chaotic setting must act under time constraints while motivated by the stakes of saving lives. Such situations typically involve the combined influence of time pressure and reward value rather than either factor alone. Understanding this interaction is essential for explaining search behavior in naturalistic settings and for developing models that better capture the complexity of visual attention allocation in real-world settings. The following sections review how time pressure and reward value have been examined in the visual search literature to date.

#### Effect of time pressure on visual search

Time pressure often results in added stress on the operator performing a task (Driskell, 2013). In the visual search literature, time pressure has been operationalized in two primary ways: by instructing participants to emphasize speed (McCarley, 2009) or by imposing an explicit time limit to complete the search task (Cambronero-Delgadillo et al., 2025; Hu et al., 2024; Nowakowska et al., 2021; Rieger et al., 2021; Slobounov et al., 2000; Sui et al., 2022; Yu et al., 2015). Among studies employing time limits, some have applied a uniform constraint across participants (e.g., a fixed 3 s limit) Rieger et al. (2021); Sui et al. (2022); Nowakowska et al. (2021), whereas others have tailored time limits based on each participant’s baseline performance under untimed conditions (Cambronero-Delgadillo et al., 2025; Hu et al., 2024; Slobounov et al., 2000; Yu et al., 2015). These paradigms have been examined across a range of visual search tasks, including applied contexts such as X-ray screening (McCarley, 2009; Rieger et al., 2021), foraging (Ries et al., 2025), as well as more abstract search tasks involving shapes (Fan et al., 2017; Slobounov et al., 2000; Yu et al., 2015) or letters (Cambronero-Delgadillo et al., 2025; Hu et al., 2024; Nowakowska et al., 2021).

Across these task domains and operationalizations, a consistent finding is that time pressure induces a speed-accuracy tradeoff. Under increased time pressure, participants typically respond more quickly but with reduced accuracy (Szalma et al., 2008). This pattern reflects a fundamental constraint of human information processing, whereby prioritizing speed limits the amount of evidence that can be accumulated before a decision is made, thereby increasing the likelihood of errors (Heitz, 2014).

Eye tracking provides a useful way to examine the processes underlying the speed-accuracy tradeoff by revealing how time pressure changes visual search behavior. In particular, eye movement measures can help distinguish how time pressure affects each of target acquisition and verification. In an X-ray screening task, McCarley (2009) found that time pressure led participants to sample images more sparsely, as reflected in shorter fixation durations and fewer but longer saccades, despite preserved target recognition once the target was fixated. Similarly, another X-ray screening study reported that search under time pressure was less exhaustive, indicated by a reduced search space (Rieger et al., 2021). Together, these findings suggest that time pressure may truncate target acquisition by limiting visual sampling, which in turn leads to reduced accuracy under time constraints.

In contrast, evidence from more abstract tasks is mixed. In a letter-based visual search task consisting of two consecutive searches, Cambronero-Delgadillo et al. (2025) found that fixation duration, fixation count, and saccade amplitude were not significantly affected by time pressure in either search phase. Moreover, Nowakowska et al. (2021) reported no effect of time pressure on search efficiency, defined as the number of superfluous fixations to non-target regions prior to making a response. This suggests that the speed-accuracy tradeoff may arise primarily during the verification phase, consistent with prior modeling work showing that speed-accuracy manipulations predominantly affect the duration of the decision stage (Rinkenauer et al., 2004).

Taken together, prior work reveals several limitations. First, existing findings do not clearly identify how each of target acquisition and verification are influenced by time pressure. This investigation could help better understand the processes underlying the speed-accuracy tradeoff. Second, eye-tracking analyses in related studies have largely relied on global metrics like fixation counts and durations, saccade amplitudes, and heatmaps. This partly reflects the use of abstract search displays in related work (e.g., arrays of letters or simple shapes). Consequently, area-of-interest (AOI) metrics like object count and mean dwell time were less applicable in these paradigms, limiting insight into object-level and semantic processing during search. Incorporating the latter can provide more interpretable measures of how attention is allocated across meaningful scene elements, yielding clearer insight into search efficiency. Third, relatively little work has examined the effects of time pressure in naturalistic visual search environments, where task factors more closely resemble that of real-world search tasks.

#### Effect of reward value on visual search

Reward value refers to the gain or loss associated with successfully completing a search task (Wolfe & Horowitz, 2017). Reward can bias visual attention through two primary mechanisms: learning and incentive motivation (Chelazzi et al., 2013). Learning-based effects have been widely studied in paradigms where reward is conditioned or primed toward specific stimuli or spatial locations (Bourgeois et al., 2018; Chelazzi et al., 2013; Hickey et al., 2011; Le Pelley et al., 2019). In such cases, reward is associated to particular objects or regions in the search space over repeated trials, often reinforced through trial-by-trial feedback (Kristjansson et al., 2010). For example, Anderson et al. (2011) used a line-orientation task in which correct responses yielded either low (1 cent) or high (5 cents) reward. They found that nonsalient, task-irrelevant stimuli previously associated with reward subsequently captured attention and slowed search performance, demonstrating involuntary value-driven attentional capture. Importantly, such learned reward biases can persist even when reward-associated stimuli conflict with current task goals, highlighting the automatic nature of learned reward biases (Failing et al., 2015; Le Pelley et al., 2019).

Incentive motivation, the mechanism of interest to the present work, refers to cases in which reward serves as a motivational signal to optimize performance (Chelazzi et al., 2013). Critically, it is not consistently tied to a particular stimulus feature or spatial location, but instead can vary across trials (Navalpakkam et al., 2010). Several studies have investigated whether incentives linked to targets that vary from trial to trial can enhance visual search performance. For example, in the second experiment by Navalpakkam et al. (2009), reward was implemented as a competitive monetary incentive: participants accumulated gains and losses across conditions, and the highest-scoring participant received a $50 bonus. The authors found an improvement in target detection performance under this reward manipulation compared to no reward. In contrast, Nowakowska et al. (2021) manipulated reward by providing additional incentives for faster responses in a letter search task, but observed no meaningful differences in search accuracy, reaction time, or search efficiency. However, prior work has largely focused on outcomes such as search accuracy and response time. As a result, it remains unclear whether incentive motivation affects target acquisition and verification, or object-based measures that capture how visual attention is allocated across the scene.

When incentive motivation does yield performance benefits, two main accounts have been proposed. One account attributes improvements to enhanced perceptual processing, whereby reward increases sensitivity to its associated stimuli through changes in neural activity within visual processing areas (Engelmann et al., 2009). A second account emphasizes strategic cognitive control, suggesting that participants allocate attentional resources to maximize expected reward. Evidence indicates that motivational and perceptual relevance can be integrated early in sensory processing to form a trial-by-trial priority representation, which guides attention toward locations associated with the highest expected value (Navalpakkam et al., 2010). Overall, incentive motivation may mobilize attentional resources to maximize reward outcomes (Chelazzi et al., 2013), and is therefore expected to modulate visual search strategies.

In addition to the presence of reward, several studies suggest that the *magnitude* of reward value may further modulate visual attention and search performance. For example, Kiss et al. (2009) associated different reward levels with target color (e.g., 5 bonus points for one color and 1 bonus point for another) and found that high-reward targets elicited faster response times as well as earlier and larger N2-posterior-contralateral (N2pc) components. The N2pc, a well-established electrophysiological marker of attentional selection, is typically observed over posterior electrodes contralateral to an attended stimulus (Eimer, 2014). Its modulation by reward magnitude suggests that incentive value enhances early attentional selection processes, biasing competition in favor of high-reward targets relative to low-reward targets. Similarly, Kristjansson et al. (2010) used a 1:10 reward ratio and offered additional monetary incentives to top-performing participants. They observed not only improved performance for the more highly rewarded target color, but also stronger trial-to-trial priming effects when high-reward targets were repeated. Together, these findings motivate examining reward magnitude beyond a binary reward manipulation, as varying reward levels may produce graded effects on attentional allocation and search efficiency.

#### The joint influence of time pressure and reward value on visual search

While prior studies have examined the effect of time pressure and reward separately on visual search (e.g., (Bourgeois et al., 2018; Nowakowska et al., 2021; Rieger & Manzey, 2022)), relatively few studies have examined how their interaction affected visual search behavior and performance. Walle and Druey (2021) postulated that reward value enhances both search efficiency and non-search processing speed, with greater benefits observed under higher task demands. However, their study did not include eye-tracking metrics, limiting insights into underlying oculomotor mechanisms. Similarly, Wagner et al. (2023) showed that under time pressure, participants were willing to incur search performance costs to maximize monetary gains. Another study reported that monetary motivation eliminated accuracy declines during dual-target searches, whereas time limits alone did not affect performance (Clark et al., 2011). Navalpakkam et al. (2010) showed that when participants searched for varying targets under time pressure, they directed their visual attention toward locations with the highest expected reward, consistent with reward-maximization behavior. Notably, this effect was observed in the absence of training or learning. Together, these findings suggest that reward value can potentially buffer some of the adverse effects of time pressure, yet the precise dynamics of their joint influence on visual search behavior and performance remain underexplored.

## The present work

The present study examines how time pressure and reward value influence visual search behavior and performance in a naturalistic setting. Specifically, we assess their effects on target acquisition and target verification—two key components of visual search that reflect how efficiently participants orient toward a potential target and how effectively they confirm its identity. In addition, we employ object-based AOI metrics such as object count and mean dwell time to capture search efficiency in a semantically rich environment.

To this end, we had forty participants search for objects of varying reward values under different time constraint conditions in a virtual living room presented in virtual reality (VR). VR has been widely used to investigate visual search behavior, with eye-tracking enabling detailed analysis of how people explore naturalistic environments (e.g., (Beitner et al., 2023; Bennett et al., 2021; Bourgeois et al., 2018; Hadnett-Hunter et al., 2019; Kit et al., 2014; Li et al., 2016, 2018; van den Oever et al., 2022)). While the present study could in principle be implemented on a 2D display, VR offers an incremental yet important advantage by supporting head movements during search in a wide field of view. This facilitates more naturalistic and ecologically valid search behavior (Jerald, 2016), while simultaneously allowing for precise gaze recording via a head-mounted eye tracker.

The central research questions of the present work are:

- How does time pressure and reward value interact to influence search performance, target acquisition and verification, and search efficiency in naturalistic visual search? Further, how does reward magnitude moderate these effects?

Based on prior work, we propose the following hypotheses:

- **H1:** Time pressure will shorten search time, target acquisition time, and verification time, and reduce object count, but at the cost of lower search accuracy (Cambronero-Delgadillo et al., 2025; McCarley, 2009; Rieger et al., 2021; Rieger & Manzey, 2022).
- **H2:** Reward value will foster more efficient search strategies, resulting in shorter search time, faster target acquisition, fewer object count, and higher accuracy (Kiss et al., 2009; Kristjansson et al., 2010).
- **H3:** Assuming time pressure will reduce search accuracy, reward value will mitigate this decline, such that accuracy under time pressure will be higher in reward trials than in no-reward trials Walle and Druey (2021); Wagner et al. (2023).
- **H4:** Higher reward magnitude will strengthen the effects predicted in H2 and H3. Specifically, relative to the low-reward condition, the high-reward condition will produce shorter search time, faster target acquisition, fewer objects inspected, and higher accuracy (H2), and will more strongly attenuate time pressure-related reductions in accuracy (H3). In turn, the low-reward condition will show greater benefits than the no-reward condition (Kiss et al., 2009; Kristjansson et al., 2010; Navalpakkam et al., 2009).

## Methods

### Participants and apparatus

An *a priori* power analysis was conducted in G*Power for a 2×3 within-subjects repeated-measures design. Assuming a medium effect size (f=.25) based on our pilot study, an α level of .05, and a desired power of .95, the analysis indicated a minimum required sample size of twenty-eight participants (Faul et al., 2007). Because this calculation is based on a simplified repeated-measures ANOVA framework, it should be interpreted as an approximate lower bound on the power of the mixed-effects models used in the present analyses. Forty undergraduate and graduate students from the University of Virginia were recruited for this study (15 female, 25 male; age range: [18, 39]; M=22.6, SD = 4.39). All participants had self-reported normal or corrected-to-normal vision. Data from one participant were discarded due to recording errors, yielding a sample size of 39 participants for data analysis purposes. Data loss from the resulting sample size after data filtering and preprocessing was 5.1%.

The VR headset used was the HTC Vive Pro Eye (1440 × 1600 pixels resolution per eye, framerate of 90 fps, nominal field of view of $110^\circ$). The built-in eye tracker was used to record participant’s gaze at 90 Hz with an accuracy of $1^\circ$ visual angle. All scenes were created using Unity 3D. The computer used to render the virtual environment had an Intel Xeon W-2225 processor at 4.10 GHz and 32 GB of RAM. The graphics card was an NVIDIA Quadro RTX 4000 (8 GB of dedicated GDDR6 memory).

### Experiment design

Participants performed a naturalistic visual search task with real-world stimuli, similar to previous work (Botch et al., 2023; Malpica et al., 2020; Olk et al., 2018). The experiment manipulated two within-subject independent variables: (1) time pressure (i.e., with time constraint, without time constraint) and (2) reward value (i.e., no reward, low reward, high reward), yielding a 2 × 3 factorial design. There were 6 blocks of 20 trials each, and all trials were target-present, with exactly one target guaranteed to appear in the scene.

Time pressure was implemented by imposing a response deadline in the timed condition. The selected time limit allowed the participants to locate any target in the room while still posing a challenge. Since search time depends on the task and experimental factors like the scene’s dimensions and scene complexity, choosing a time limit should be based on the present experiment. Based on a pilot study with six participants, the mean search time was 1.97 s $(95\%\text {CI}=[1.64,2.30])$. Thus, a 3-s time limit was established for the timed condition.

Reward value was operationalized as the number of points participants could earn for successfully locating the target within a trial. Low- and high-reward trials were worth 1 and 10 points, respectively, whereas no-reward trials provided no points, following prior reward-based visual search paradigms (Bourgeois et al., 2018; Kristjansson et al., 2010). Reward values were intermixed within each block rather than presented in separate reward-specific blocks to prevent participants from selectively prioritizing effort during high-reward periods.

### Experimental procedure

Participants first read a debrief, signed a digital consent form, and were asked to remain seated in a stationary chair. They put on the VR headset and completed the built-in eye-tracking calibration and validation procedure provided by HTC. Participants watched a slideshow of all objects and their names at the beginning of the experiment to eliminate any possible confusion in finding the right object (Le-Hoa Võ & Wolfe, 2015). Objects in the slideshow were presented in orientations consistent with how they would appear in a living room environment (Vickery et al., 2005). Next, the experimental software developed for this study was launched on the VR headset and participants completed a training block. Each trial then began with participants fixating on a central red sphere for 3 s for the target information to load. The display provided instructions on what to find (i.e., target name), the reward value for finding the target, and whether there was a time limit or not. Afterward, the participants fixated on the center of the text for another 3 s. Then, the virtual living room loaded onto the virtual headset’s display, and participants began their search for the target.

**Fig. 2:**
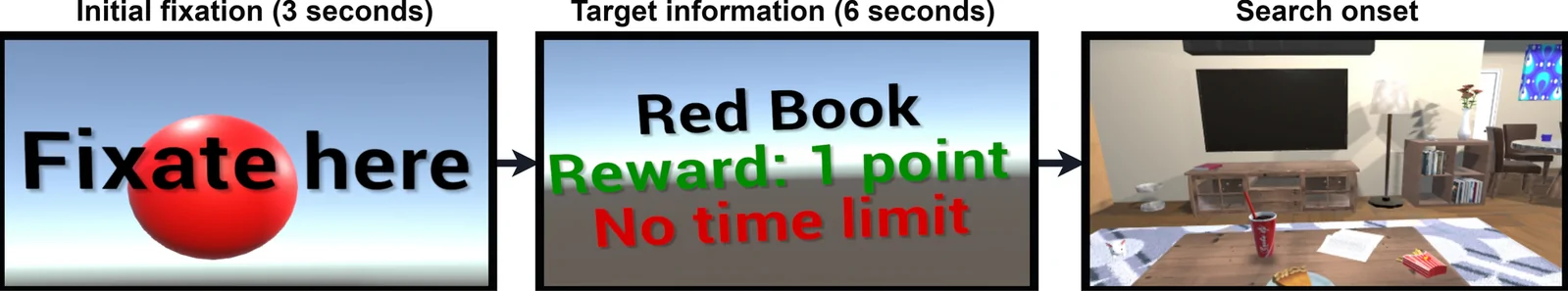
Sequence of events the participants experienced for each trial

If participants located the target, they were instructed to fixate on it and press the trigger on the right-hand controller to indicate detection. A trial was considered correct if the participant fixated the target at the time of the trigger press, thereby ending the trial. To account for cases in which participants identified the target but briefly shifted their gaze immediately before responding, such instances were manually investigated and corrected during preprocessing. If the participant failed to find the target within the time limit, the trial automatically ended and a new trial commenced. In other words, participants were not allowed to continue their search past the time limit. The sequence of events is summarized in the block diagram in Fig. 2.

Participants were not given feedback after the trial to prevent reinforcement effects. Participants could give up at any time for any reason by clicking the trigger. Neither time limit nor the reward value was visually displayed to the participants while they searched so it would not divert their gaze from the task. The entire experiment took 45 min, and participants were compensated $15 for their time. To encourage participants to collect rewards, the top three performing participants with most reward points collected were awarded an additional $100, $75, and $50, respectively. Participants were not informed of their reward count throughout the experiment. Participants were not told to find the target as quickly as possible, meaning they could devise a speed or accuracy-driven strategy in the untimed condition.

### Scene development and design considerations

**Fig. 3:**
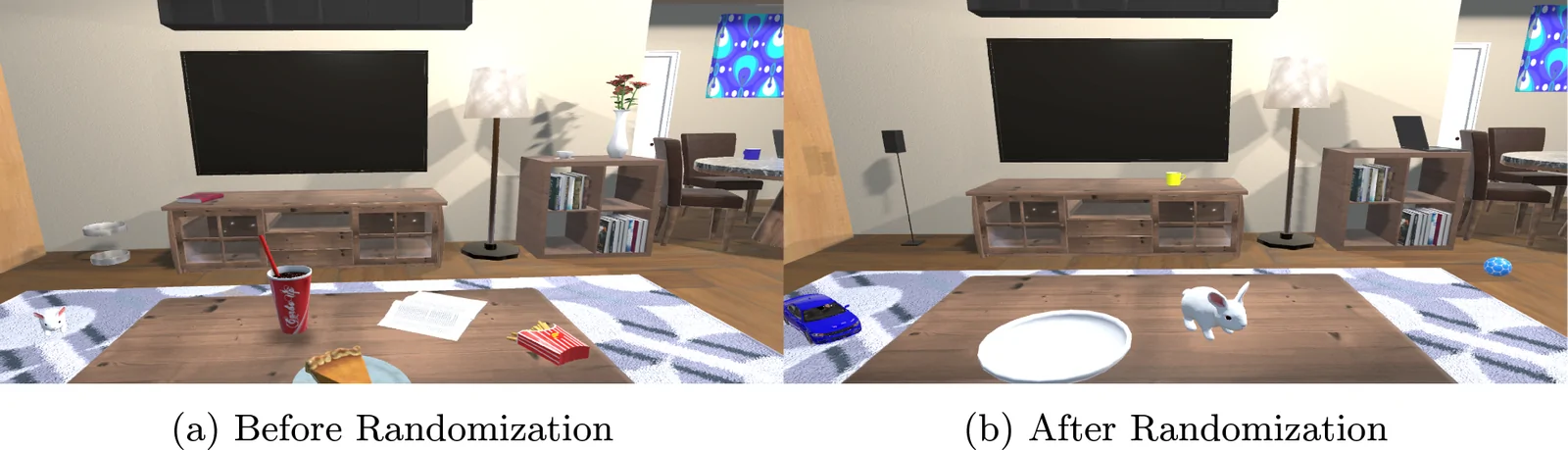
Two views of the living room demonstrating how the objects and their locations were randomized each trial

Each trial consisted of 24 unique objects arranged in the virtual living room, with one designated as the target. To ensure diversity in both visual appearance and semantic category, the object set spanned four color classes (i.e., red, blue, green, and white) and six object classes of everyday living room items (i.e., mugs, toys, food items, pillows, books, and vases). Target identity and reward value order were randomized for each participant. Targets could appear on semantically appropriate surfaces in the living room, including the floor (e.g., a floor lamp), shelves (e.g., a vase), or tables (e.g., a coffee cup). Placement was constrained by object semantics (e.g., books could appear on multiple surfaces, whereas floor lamps could only appear on the floor); therefore, the set of objects available on each surface was held constant. An example of the location randomization is shown in Fig. 3.

To control for search history effects (Wolfe, 2020; Wolfe & Horowitz, 2017) and preserve environmental novelty, object locations were randomized on every trial. No object or target appeared on the same surface on two consecutive trials. The living room provided a $180^\circ$ field of view, requiring participants to move their heads to locate targets beyond the headset’s nominal $110^\circ$ view. Randomization also mitigated spatial biases and target eccentricity effects (Le-Hoa Võ & Wolfe, 2015) by spawning targets at varying distances from central fixation with equal probability.

### Gaze data collection

**Fig. 4:**
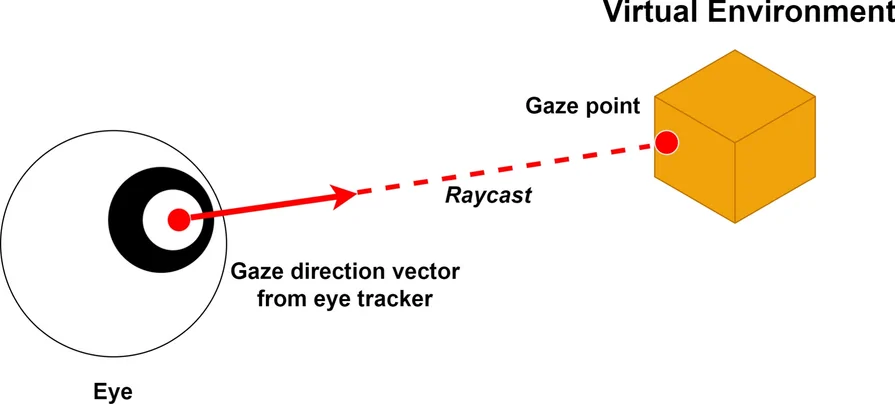
Illustration of a Raycast colliding with a virtual cube

User gaze was captured using the built-in eye tracker of the HTC headset and Unity. The SRanipal software development kit (SDK) developed by HTC for the built-in eye tracker allows for the collection of pupil diameter, gaze origin, and gaze direction vector per eye along other measures. Using the coordinates of the gaze direction vector, we use the Raycast function in Unity to project a ray from the pupil. If this ray collides with an object in our virtual environment, we know the user is looking at it. In Fig. 4 for example, the ray collides with a cube at a point which acts as our focus point. The game engine allows us to extract the collision point coordinates, enabling us to record the user’s gaze in 3D space.

To account for the eye tracker’s angular error when coding object fixations, we increased the object collider sizes. In Unity, colliders are components used for physical interactions and collision detection. Based on pilot testing, each collider was set to be approximately 10% larger than the corresponding object mesh. This adjustment was intended to accommodate two main sources of error: (1) the manufacturer-reported minimum angular error of the eye tracker, and (2) variation in viewing distance, such that objects farther from the participant subtend smaller visual angles. Under these conditions, participants could be fixating an object even if the gaze ray did not consistently intersect the object’s exact collider.

More generally, this approach reflects a limitation of modeling gaze as a single ray (e.g., via raycasting), which misrepresents the functional field of view. Expanding the collider therefore reduced edge cases in which fixating on the target might otherwise be missed. Pilot observations showed that such missed-collision cases were effectively mitigated after expanding the collider size.

The gaze data collection script was coded using C# programming. Although the HTC Vive Pro Eye tracker is advertised to run at a maximum frequency of 120 Hz, it can only run at 90 Hz while using the functionalities described above due to technical constraints between Unity and the SRanipal SDK.

### Metrics

Recall that, in the timed condition, trials terminated after 3 s, thus decreasing accuracy if the target was not found within the time window. Although the 3 s limit was selected based on pilot testing to allow sufficient time to complete even the most difficult searches, some trials nevertheless could not be completed under time pressure. This creates an imbalance in the composition of correct trials: more difficult searches remain included in the untimed condition, where participants could exceed 3 s, but are excluded from the timed condition once the time limit is reached. As a result, apparent differences between conditions could reflect differences in trial composition rather than genuine behavioral changes induced by time pressure.

To address this concern, we restricted the primary analysis to trials completed within 3 s (86.19% of all trials). Trimming at 3 s ensures that timed and untimed conditions are compared over a common set of feasible trials and reduces confounds arising from differences in trial inclusion. Under this approach, any observed effects can be more confidently attributed to changes in visual search behavior due to time pressure rather than to artifacts of the imposed time limit (see (Adamo et al., 2019) for a similar rationale). Next, we outline the metrics, grouped into search performance and behavior.

#### Search performance

- **Search accuracy.** The ratio of correct to total trials per block. Accuracy was not restricted to searches within the 3 s limit in the untimed condition. Higher accuracy indicates better performance.
- **Search time.** The time between the trial onset and the end of the trial. Lower search time indicates better performance.

#### Search behavior

- **Target latency.** Also known as the time to first fixation (Nowakowska et al., 2021) or elapsed time to target fixation (Horstmann et al., 2019), target latency is the time between the onset of the search and the first fixation on the target (Castelhano & Heaven, 2010). Lower target latency suggests less time to acquire the target.
- **Verification time.** The time between the first fixation on the target and the onset of the trigger click. Verification time indicates the time to process and verify the target (Castelhano & Heaven, 2010). Lower verification time suggests less time to process the target.
- **Object count.** Object count was defined as the number of unique AOIs gazed upon within a trial. This measure is distinct from fixation count, as a given object may be fixated on multiple times. Thus, object count provides insight into the thoroughness of the search and how visual attention is distributed across different AOIs Objects included any of the 24 objects in the scene as well as non-target surfaces such as tables, walls, and shelves. Given that the analysis was restricted to correct trials, a lower object count indicates a more efficient search strategy.
- **Mean dwell time.** The mean of the times spent dwelling on each object (Borys, 2017; Horstmann et al., 2019). Mean dwell time was measured in ms. Given that the analysis was restricted to correct trials, a shorter mean dwell time suggests a more efficient search strategy.

### Statistical analysis

Data preprocessing was performed following data collection. First, missing and invalid data entries were excluded. The SRanipal SDK provides validity indicators, such as eye_valid and per-eye openness, to assess data quality. According to the SDK specifications, samples with an eye_valid value below 31 are considered invalid and were therefore omitted. After preprocessing, 3,518 correct trials remained for statistical analysis.

The effects of time pressure and reward value on each of our metrics were assessed using linear mixed models (West & Welch, 2014) implemented using the *lme4* package (Bates et al., 2014; Winter, 2013). Continuous variables were fitted with Gaussian distribution models, while discrete variables (fixation count, saccade count, and object count) were fitted with Poisson distribution models. In case of overdispersion, negative binomial distribution models were used. The models for each metric were fitted using full maximum likelihood estimation following a stepwise forward procedure, where a model first starts with the random intercepts for participants then fixed effects are added one at a time for the two predictor variables and their interaction. Likelihood ratio tests were then used to determine if adding each factor significantly improved the model fit. The p values for these tests were adjusted using the Benjamini–Hochberg procedure (Benjamini, 1995), a balanced approach to control family-wise error rate upon performing multiple comparisons. Significant effects were further explored using Tukey post hoc comparisons. Residual QQ plots were visually inspected for assumption violations. All analyses were conducted in R (Version 4.3.2; (Clark et al., 2024)), with estimated marginal means and degrees of freedom calculated following established methods (Bates et al., 2014; Lenth, 2024).

## Results

We begin by reporting search performance, including search accuracy and search time, followed by search behavior, which comprise target latency, verification time, object count, and mean dwell time. Means and standard deviations are reported in Tables 1, 2, and 3 in the Appendix.

### Search performance

#### Search accuracy

Time pressure had a significant main effect on accuracy $(\chi ^2(1) = 91.53, p <.001)$, where the untimed condition was more accurate than the timed condition (mean difference = 10.81%, t=11.29, p<.001). Reward value also had a significant main effect on accuracy $(\chi ^2(2) = 8.34, p =.048)$, where the no-reward condition was less accurate than the low-reward (mean difference = 4.13%, t=3.51, p=.001) and the high-reward conditions (mean difference = 3.74%, t=3.18, p=.004). Accuracy did not differ between the low- and high-reward conditions (mean difference = 0.390%, t=0.338, p≥.938).

There was a significant time pressure × reward value interaction effect on accuracy $(\chi ^2(2) = 7.83, p =.049)$, indicating that the effect of reward on accuracy depended on time pressure. Specifically, in the timed condition, accuracy was higher in the low-reward condition (mean difference = 7.25%, t=6.10, p<.001) and the high-reward condition (mean difference = 6.20%, t=3.71, p=.003) relative to the no-reward condition. In contrast, in the untimed condition, accuracy did not differ significantly across reward levels (all mean differences ≤ 1.00%, all p≥.989).

#### Search time

There was no significant time pressure × reward value interaction effect on search time $(\chi ^2(2) =.283, p =.867)$. Time pressure did not significantly influence search time $(\chi ^2(1)=.617, p=.864)$. Recall that only trials completed within 3 s were included in the primary analysis. Including trials exceeding 3 s yielded a significant main effect of time pressure on search time $(\chi ^2(2)=301.84, p<.001)$, with shorter search times in the timed condition than in the untimed condition (mean difference = .553 s, t=17.69, p<.001). However, trials exceeding 3 s may reflect more difficult searches that could not be completed under time pressure and were therefore disproportionately excluded from the timed condition. For instance, targets in trials exceeding 3 s could have been located farther from the initial point of fixation and therefore could not be identified within the time limit. This interpretation is supported by the larger mean visual angle in trials exceeding 3 s $(M = 1.24^\circ )$ compared with trials completed within 3 s $(M = 1.03^\circ )$.

**Fig. 5:**
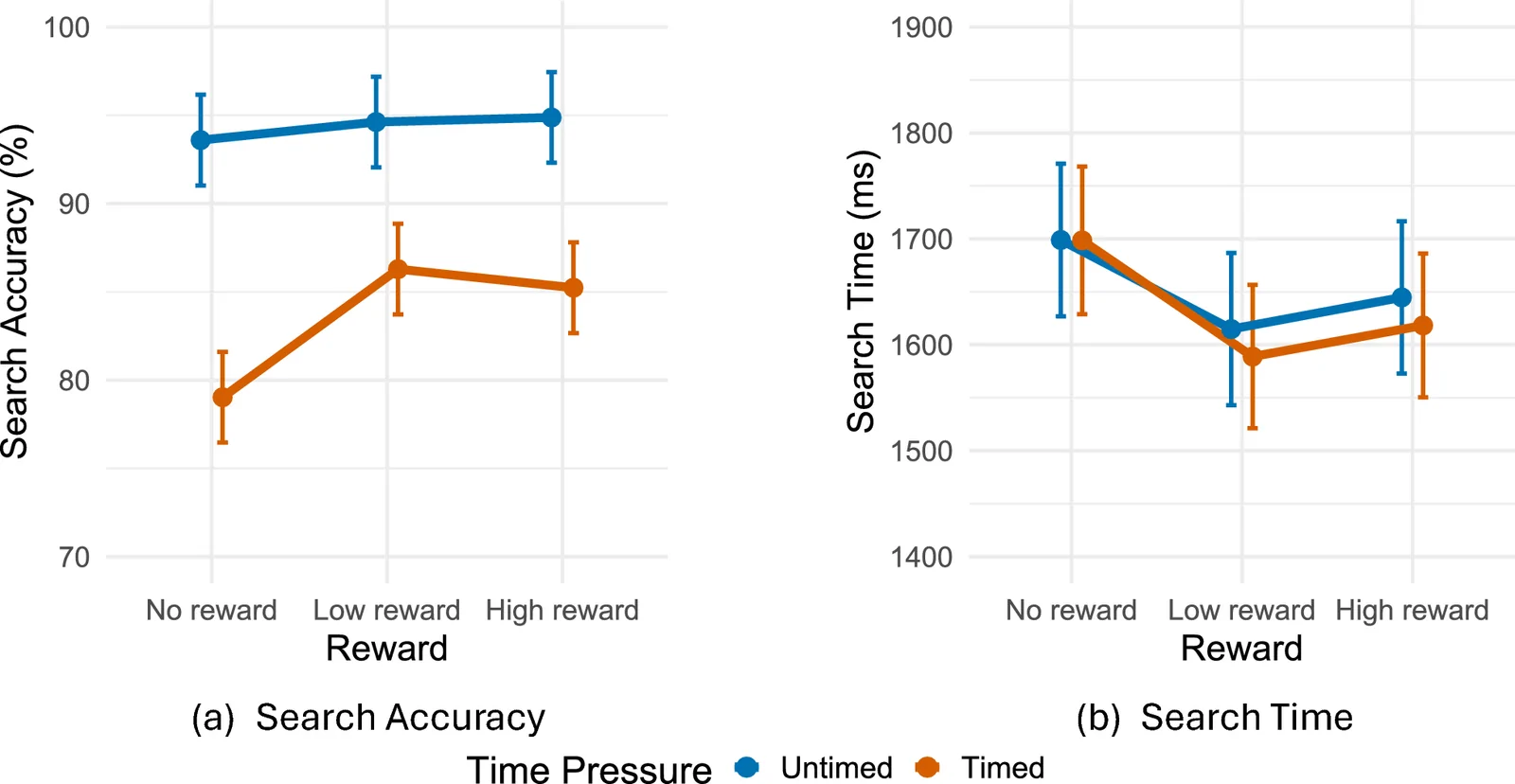
Line plots showing the marginal mean values and 95% confidence intervals for **a** search accuracy and **b** search time as a function of time pressure and reward value

In contrast, reward value significantly influenced search time $(\chi ^2(2) = 13.02, p =.007)$. Search time was longer in the no-reward condition than in both the low-reward condition (mean difference = 96.88 ms, t=3.47, p=.001) and the high-reward condition (mean difference = 77 ms, t=2.41, p=.042). There was no significant difference in search time between the low- and high-reward conditions (mean difference = 29.60 ms, t=1.08, p=.528). These results are illustrated in Fig. 5.

### Search behavior

#### Target latency and verification time

There was no significant time pressure × reward value interaction effect on target latency $(\chi ^2(2) =.197, p = 1.00)$. Time pressure significantly influenced target latency $(\chi ^2(1) = 8.12, p =.039)$, with longer latencies in the timed condition compared with the untimed condition (mean difference = 65.63 ms, t=2.86, p=.039). Reward value also significantly influenced target latency $(\chi ^2(2) = 10.08, p =.050)$. Specifically, target latency was longer in the no-reward condition than in the low-reward condition (mean difference = 90.15 ms, t=3.20, p=.003), with no significant differences between the remaining reward conditions. There was no significant time pressure × reward value interaction on target latency $(\chi ^2(2) =.203, p = 1.00)$.

**Fig. 6:**
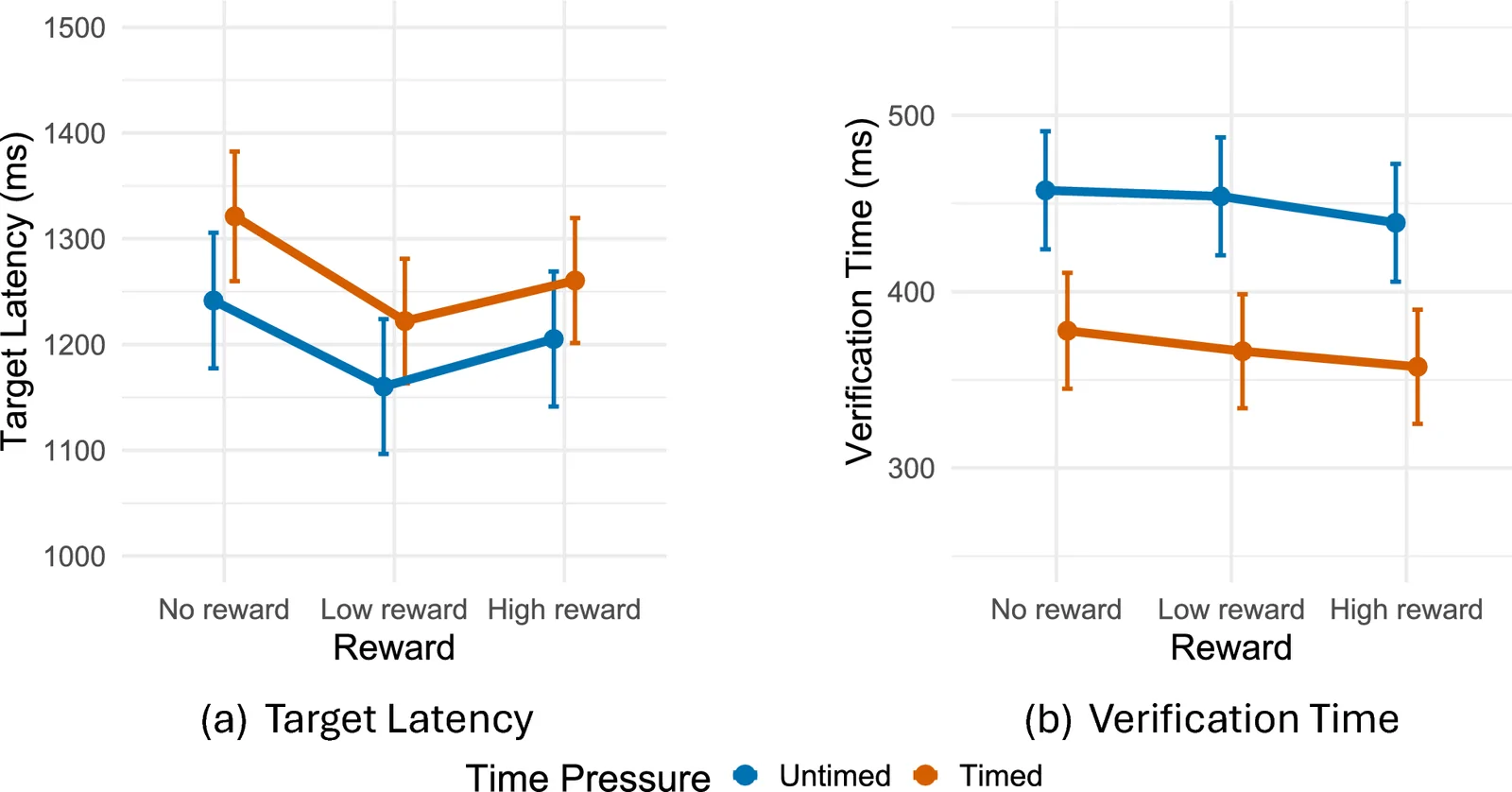
Line plots showing the marginal mean values and 95% confidence intervals for **a** target latency and **b** verification time as a function of time pressure and reward value

There was no significant time pressure × reward value interaction effect on verification time $(\chi ^2(2) =.283, p =.867)$. Time pressure significantly influenced verification time $(\chi ^2(1) = 110.40, p <.001)$, with shorter verification times in the timed condition compared with the untimed condition (mean difference = 83.07 ms, t=10.57, p<.001). Reward value did not significantly influence verification time $(\chi ^2(2) = 4.74, p =.652)$. The results are shown in Fig. 6.

#### Object count and mean dwell time

There was no significant time pressure × reward value interaction effect on object count $(\chi ^2(2) =.197, p = 1.00)$. Time pressure did not significantly affect object count $(\chi ^2(1) = 2.60, p =.652)$. In contrast, there was a significant main effect of reward value on object count $(\chi ^2(2) = 18.61, p <.001)$, where the no-reward condition had a higher object count than both the low-reward condition (mean difference = .331 objects, t=4.14, p<.001) and the high-reward condition (mean difference = .253 objects, t=3.16, p=.004), with no difference between the low- and high-reward conditions (mean difference = .100 objects, t=.996, p=.579).

**Fig. 7:**
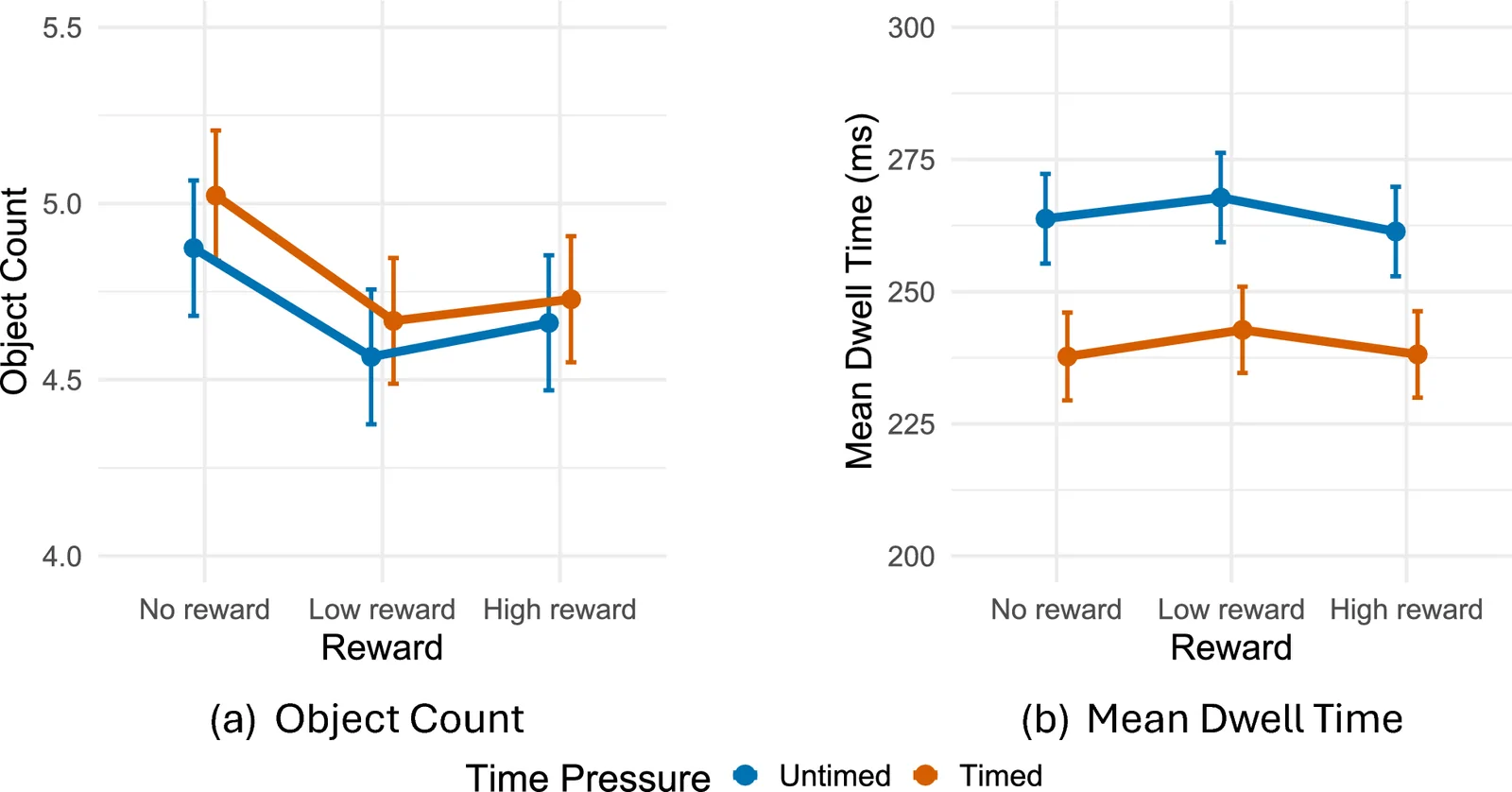
Line plots showing the marginal mean values and 95% confidence intervals for **a** object count and **b** mean dwell time as a function of time pressure and reward value

There was no significant time pressure × reward value interaction effect on mean dwell time $(\chi ^2(2) =.264, p = 1.00)$. Time pressure significantly influenced mean dwell time $(\chi ^2(1) = 133.04, p <.001)$, where the timed condition had shorter mean dwell times (mean difference = 24.70 ms, t=11.65, p<.001) than the untimed condition. On the other hand, reward value had no significant effect on mean dwell time $(\chi ^2(2) = 4.62, p =.652)$. The results are shown in Fig. 7.

## Discussion

The present study investigated the impact of time pressure, reward value, and their interaction on naturalistic visual search behavior in VR. We discuss our findings in relation to our search performance and behavior metrics.

### Search performance

Time pressure significantly reduced search accuracy, consistent with prior work demonstrating accuracy detriments under temporal constraints (McCarley 2009; Rieger et al. 2021; Rieger and Manzey 2022). In contrast, reward value improved accuracy, aligning with evidence that incentives can enhance performance in visual search tasks (Kiss et al., 2009; Kristjansson et al., 2010; Walle & Druey, 2021). Notably, accuracy was the only outcome measure in the present study to exhibit a significant time pressure × reward value interaction.

Specifically, participants were more accurate under time pressure when targets were associated with reward. This finding supports our hypothesis (H3) that reward can mitigate the performance costs induced by limited search time and is consistent with previous work reporting similar interaction patterns (Clark et al., 2011). Importantly, reward value did not significantly affect accuracy in the untimed condition (Fig. 5a). This may reflect a ceiling effect, where participants could achieve high accuracy regardless of reward value in the absence of a time constraint. This interpretation is consistent with prior studies showing that reward-related benefits become more pronounced as task demands increase and may be absent in easier search conditions without time pressure (Walle & Druey, 2021), as well as with broader accounts suggesting that some easy search tasks already operate at highly efficient or near-optimal levels (Wolfe, 2020). This interpretation is also consistent with the high accuracy observed in the untimed condition (around 93%). Future work employing more complex or demanding search environments may help determine whether reward value also yields measurable accuracy benefits in untimed contexts.

However, contrary to prior findings suggesting graded benefits with increasing reward value (Bourgeois et al., 2018; Kristjansson et al., 2010), reward magnitude did not further moderate this effect. Although both low- and high-reward conditions yielded higher accuracy than the no-reward condition, performance did not differ reliably between the two reward values. Thus, the present results do not provide evidence in support of H4. It is unlikely that the choice of reward values alone explains this null effect, as similar reward ranges have produced significant effects in prior work (Bourgeois et al., 2018). We consider two possible explanations. First, the present search task was relatively brief and simple, which may have limited the additional benefits of increasing reward magnitude beyond the presence of reward itself. Second, incentive effects may plateau or even become counterproductive at higher levels, such that larger rewards do not necessarily yield proportional performance gains (Ariely et al., 2009). Further work using more demanding search contexts and a broader range of reward values is needed to verify any of these explanations.

For search time, analyses including trials exceeding 3 s indicated that time pressure reduced search time. Coupled with the observed decrease in accuracy, this pattern is consistent with a speed-accuracy tradeoff, whereby faster search time under time pressure comes at the cost of reduced accuracy. However, as discussed in the Methods section, this apparent reduction in search time may largely reflect a compositional artifact. It could be that time pressure prevented participants from completing the most difficult searches. For this reason, the trimmed analysis restricted to trials completed within 3 s provides a more informative comparison, as it evaluates whether time pressure alters search speed even among feasible, successfully completed trials. Within this restricted set, time pressure did not significantly reduce search time, contrary to prior studies reporting faster searches under time constraints (McCarley, 2009; Rieger et al., 2021; Rieger & Manzey, 2022; Slobounov et al., 2000).

This suggests that time pressure primarily influenced whether difficult searches could be completed successfully, rather than accelerating search speed on correct trials. For example, under time pressure, if a participant initially directed their search toward the wrong region of the living room (e.g., the far right side when the target was on the far left), there was limited opportunity to reorient and correct this strategy before time ran out. Such trials were more likely to end in an error because the target could not be located in time. Importantly, time pressure did not cause the participant to scan the chosen region more quickly; rather, it reduced the ability to recover from incorrect search trajectories.

One might claim that the absence of faster search time under time pressure reflects the brevity of the task itself, leaving limited room for further reductions in completion time. This claim is unlikely, as search times were influenced by reward value, with participants completing searches more quickly in rewarded conditions than in the no-reward condition. The findings support H2, where reward value improved both accuracy and search speed. Thus, this contrast suggests that time pressure—implemented here as a hard response deadline—alone does not necessarily lead to faster search on correct trials, but instead primarily constrains the ability to successfully complete more difficult searches within the imposed time limit.

### Search behavior

#### Target latency and verification time

No significant time pressure × reward value interactions were observed for target latency or verification time; therefore, we discuss the effects of time pressure and reward value separately for these measures.

Regarding target acquisition, participants took longer to acquire the target compared to the untimed condition. In contrast, target verification was shorter under time pressure, suggesting that once the target was fixated, participants confirmed its identity more quickly. Contrary to prior claims that time pressure uniformly reduces search and processing speed (McCarley, 2009; Walle & Druey, 2021), our results suggest that time pressure had distinct effects on each of target acquisition and verification. As discussed earlier, total search duration on correct trials remained comparable between timed and untimed conditions. However, participants under time pressure exhibited a different temporal profile: they spent longer acquiring the target, but less time verifying the target once fixated. In other words, time pressure led to a redistribution of processing time, with slower acquisition alongside faster verification, rather than a uniform speeding of both components. This discrepancy from previous studies may partly reflect differences in how time pressure was operationalized. Whereas prior work induced time pressure primarily through instructional emphasis (McCarley, 2009), the present study implemented time pressure as a time limit. Future work should directly compare these approaches to determine how different implementations shape target acquisition and verification. Overall, the present findings provide only partial support for H1, showing that time pressure decreased accuracy and verification time but unexpectedly increased acquisition time, highlighting a more nuanced account of how time pressure shapes visual search behavior.

As for reward value, participants acquired targets more quickly when they were associated with reward. However, reward value did not significantly affect verification time. Thus, reward value primarily improved the efficiency of locating targets, while the subsequent verification process remained unchanged. The reduction in overall search time (Fig. 5a) was therefore driven by faster target acquisition. These findings support H2, indicating that reward value enhances accuracy and promotes more efficient search without altering verification processes. We next examine search efficiency measures to provide a more granular account of how these changes manifested in eye movements.

#### Object count and mean dwell time

Time pressure did not significantly affect object count, but it reduced mean dwell time. However, recall that participants showed longer target acquisition under time pressure. Thus, a longer acquisition time with similar object count but shorter dwell times suggests that time pressure altered the composition of target acquisition, increasing transition time between objects while reducing the amount of information processed from each object (Holmqvist et al., 2011). This shift may have contributed to the observed decrease in accuracy, as participants extracted less information from scene elements, making target identification more difficult.

Participants scanned fewer objects when the target was associated with reward, indicating a more efficient search strategy (Navalpakkam et al., 2010). Reward value did not significantly affect mean dwell time, suggesting that participants sampled similar amounts of information from each object despite inspecting fewer objects overall. This observation helps explain why reward reduced target acquisition time while leaving verification time unchanged.

Unlike prior work reporting null effects of reward on eye gaze (James et al., 2019), our findings show that reward can alter eye movements, consistent with the broader reward-attention literature (Bourgeois et al., 2018; Failing et al., 2015; Le Pelley et al., 2019). Previous studies have shown that reward priming facilitates attentional allocation toward target features and away from distractors (Hickey et al., 2011). Importantly, in the present task, target features varied from trial to trial and object locations were randomized, such that reward effects could not be explained by learned feature or spatial associations. Nevertheless, reward still promoted more efficient attentional guidance, reducing object sampling without compromising accuracy. Finally, while the presence of reward improved search behavior, reward magnitude did not yield additional benefits within the range tested here.

Our distinction between target acquisition and verification can also be situated within classic guided search and serial–parallel accounts of visual attention. Given the heterogeneous, semantically rich search scene and the trial-by-trial variation in target identity, search in the present task is unlikely to rely on purely parallel, feature-based pop-out. Instead, the results are consistent with a guided search framework in which attention is directed by a priority map that integrates physical salience, task goals, and reward-driven value (Chelazzi et al., 2013; Wolfe, 2020). Within this account, reward value may increase the priority of target-relevant information (Navalpakkam et al., 2010), thereby improving acquisition efficiency, as reflected in shorter target latency and fewer objects inspected. In contrast, time pressure appears to alter the serial deployment of attention across objects, trading thoroughness for speed by reducing both time spent on verification and accuracy. Thus, rather than uniformly accelerating all stages of processing, time pressure and reward may differentially shape the priority map and subsequent attentional selection dynamics during naturalistic search.

### Implications

The present findings offer several conceptual implications for the design of visual search applications and models of visual attention. First, incentive-based rewards may provide a potential avenue for mitigating performance decrements under time pressure, suggesting that motivational cues could help sustain accuracy in temporally constrained search. However, these implications should be interpreted as proof-of-concept, as the current task was brief, relatively simple, and conducted within a controlled VR environment. Substantial intermediate work is required before reward-based interventions could be considered in operational contexts, including validation in more complex and domain-specific tasks, and a critical assessment of how incentives interact with fatigue, stress, and real-world consequences. Moreover, reward and gamification approaches may introduce risks in safety-critical settings, such as encouraging speed over caution or unintended strategic tradeoffs, underscoring the need for context-sensitive evaluation (Hess, 2017).

Second, the distinct eye movement patterns observed under time pressure and reward value underscore eye-tracking’s potential in detecting subtle shifts in attentional strategies driven by task-related factors. This makes eye-tracking technology a promising tool for developing context-aware adaptive systems that can respond in real-time to changing user states. For example, a driver-assistance system could monitor gaze patterns to detect when a driver begins scanning less broadly and making longer saccades—signals of a rushed search—and proactively highlight critical road signs. More broadly, such applications suggest that eye-tracking can serve as a foundation for adaptive technologies that help detect stress and sustain search performance. At the same time, significant work remains to determine which gaze-based measures are most reliable for capturing these contextual shifts in real-time, highlighting an important direction for future research.

### Limitations and future work

While the present work provided a thorough analysis of visual search behavior and performance in VR, it could be improved in several ways. First, the task was relatively short in duration and simple in complexity. As a result, the variation in findings compared to previous literature (McCarley, 2009; Sui et al., 2022) could be due to the nature of the task in the present study. Future work must investigate how time pressure and reward value affect visual search over prolonged periods and in more complex tasks. Additionally, the present work examined time pressure only in a binary manner (time pressure vs. no time pressure), given the relatively simple search task and the short search times. Future work could investigate a broader range of time limits in more complex search tasks to provide a more detailed characterization of how varying degrees of time pressure influence visual search. Additionally, the findings should be generalized to mediums other than VR with caution, and further research is necessary to determine whether the observed effects of time pressure and reward value hold in more applied VR contexts i.e., exploring a menu or playing a game. Moreover, the study’s sample size and composition might not represent the broader population, potentially limiting the external validity of the results. Thus, including a more diverse group of participants in future research could provide a more comprehensive understanding of how these variables influence visual search behavior. In addition, future work could implement more advanced eye-tracking analyses (e.g., scanpath analysis; (El Iskandarani et al., 2023)) to obtain different insights on visual search strategies. Finally, participants only had to find one target per trial in our study; it would be interesting to see how the reward value effect scales in a multiple target search task; would the fewer number of objects encoded still benefit performance in such a task or compromise it?

## Conclusion

This study examined how time pressure and reward value influence target acquisition, verification, and search performance in a naturalistic VR environment. Using object-based eye-tracking metrics, we found that time pressure and reward value exert distinct effects on search behavior. Time pressure prolonged target acquisition, shortened verification, and reduced accuracy, whereas reward value promoted more efficient search through faster acquisition and reduced object scanning. Importantly, reward partially mitigated the accuracy costs of time pressure. Overall, these findings suggest that time pressure and reward value modulate different components of visual search, with time pressure disrupting early acquisition processes and reward enhancing guidance toward targets.

## Open practice statement

The dataset used for statistical analysis is publicly available on the Open Science Framework (https://osf.io/v95dw/overview?view_only=42be19dfb92748868c7b30173d26710e). Other data and materials used in this study are available upon request from the authors. The experiment reported in this study was not preregistered.

## Appendix

See Tables 1, 2, and 3.

**Table 1:** Mean (SD) performance measures by reward value and time pressure

Reward	Time pressure	Search accuracy (%)	Search time (s)
No reward	Untimed	93.59 (6.58)	1.69 (0.66)
No reward	Timed	79.03 (10.62)	1.70 (0.70)
Low reward	Untimed	94.62 (5.43)	1.60 (0.66)
Low reward	Timed	86.28 (8.64)	1.59 (0.70)
High reward	Untimed	94.87 (5.68)	1.63 (0.66)
High reward	Timed	85.23 (10.21)	1.62 (0.70)

**Table 2:** Mean (SD) target acquisition and verification times by reward value and time pressure

Reward	Time pressure	Target latency (s)	Verification time (s)
No reward	Untimed	1.24 (0.65)	.449 (0.27)
No reward	Timed	1.32 (0.72)	.375 (0.25)
Low reward	Untimed	1.16 (0.65)	.447 (0.27)
Low reward	Timed	1.22 (0.72)	.368 (0.23)
High reward	Untimed	1.20 (0.62)	.428 (0.24)
High reward	Timed	1.26 (0.71)	.357 (0.21)

**Table 3:** Mean (SD) search efficiency measures by reward value and time pressure

Reward	Time pressure	Object count	Mean dwell time (s)
No reward	Untimed	4.86 (1.95)	.261 (0.07)
No reward	Timed	5.02 (1.97)	.237 (0.05)
Low reward	Untimed	4.54 (1.89)	.265 (0.08)
Low reward	Timed	4.67 (2.06)	.242 (0.06)
High reward	Untimed	4.64 (1.88)	.258 (0.07)
High reward	Timed	4.72 (1.92)	.238 (0.06)
